# Mucoadhesive micelles for ophthalmic drug delivery

**DOI:** 10.1177/08853282251386004

**Published:** 2025-10-07

**Authors:** Taylor Goostrey, Mitchell Ross, Karim Soliman, Lindsay Sheardown, Heather Sheardown

**Affiliations:** 1 Department of Chemical Engineering, McMaster University, Hamilton, ON, Canada

**Keywords:** nano-micelles, mucoadhesion, dry eye disease, controlled drug release

## Abstract

The most common formulation for treating ocular conditions is topical eyedrops, despite their well-documented inefficiency. In this study, mucoadhesive nano-micelles were developed to overcome the poor efficacy of topical eyedrops in the treatment of dry eye disease. The micelles contained a pre-activated thiomer capable of releasing mucolytic N-acetylcysteine upon covalent disulfide exchange with the natural mucus layer which covers the surface of the eye. The micelles, approximately 70 nm in diameter, were shown to be mucoadhesive through zeta potential analysis. The critical micelle concentration was determined to be 217 mg/L using the pyrene fluorescence method. The core of the micelles was loaded with cyclosporine A, displaying a greater than 90% entrapment efficiency, and yielding sustained release of approximately 57% over 10 days. The cellular response to the micelles was tested with human corneal epithelial cells by MTT assay and Live/Dead staining. It was found that lower concentrations of the amphiphilic polymer resulted in greater cellular viability and in all cases, viability increased from 24 to 48 h following treatment. Overall, these mucoadhesive systems have potential to provide more efficacious treatment of anterior segment ocular conditions.

## Introduction

Dry eye disease (DED) is one of the most common surface ocular disorders with global estimates of prevalence ranging from 10%–30% of the population.^1^ Common risk factors associated with DED include contact lens wear, environmental exposure, Asian ethnicity, sex, and aging.^2^ Anatomically, the tear film is comprised of an anterior, thin (approximately 80 nm) outer layer of lipids produced by the meibomian glands, followed by an approximately 3 µm thick aqueous layer containing secreted enzymes and soluble mucins, and then a layer of bound mucus which covers the corneal epithelial surface.^3^ It is estimated that approximately 10% of DED is caused by reduced tear production, while greater than 80% is caused by meibomian gland dysfunction.^4^ Individuals with DED suffer from discomfort, blurred vision, light sensitivity, and the disease can lead to damage of the corneal epithelium. DED is thought to be caused by an inflammatory cytokine process which affects the corneal surface and lacrimal gland.^5^ Therefore, the treatment of DED typically involves the application of anti-inflammatory eyedrops. Of the anti-inflammatory eyedrops, the non-steroidal, hydrophobic cyclosporine A (cycA) is the most commonly used and is prescribed to 48.2% of patients suffering from chronic DED.^6^ However, in addition to the low efficacy of topical eyedrops, with less than 5% of an applied dose reaching target tissues due to the natural clearance mechanisms of the eye, cycA eyedrops can lead to a stinging/burning sensation, which can limit patient compliance, especially with the need for frequent reapplication and require prolonged treatment before any effects are noted.^7–9^

To overcome the issues associated with topical eyedrops, various biomaterials have been developed as sustained release formulations. Of these, polymer nanoparticles (NPs) are particularly promising for anterior ophthalmic application because of their potential to increase residence time, as well as high loading of poorly water soluble drugs, and greatly increased drug penetration in ocular tissues.^10,11^ For hydrophobic cycA, copolymers that can self assemble into micelles, trapping the compound within the hydrophobic core, represent a particularly promising platform.^12^ Although micelles do improve tear film residence time compared to conventional eyedrops, they remain susceptible to washout due to tear lacrimation and tear turnover. To overcome these issues, NPs have been developed that can anchor themselves to the natural mucosal membrane of the eye thus prolonging material residence time and subsequent drug release.^13^

Several theories have been used to describe mucoadhesion of NPs with ocular surface mucins, the three most prevalent of which are (1) *adsorption*, (2) *diffusion*, and (3) *electronic interaction*.^14–19^ Various NP formulations have been developed that are capable of associating with mucin, including hyaluronic acid-based materials that hydrogen bond with mucin,^20,21^ chitosan-based materials that ionically interact with mucin,^22^ or boronic acid,^13^ thiol,^23,24^ or thiomer-based materials^25^ that can covalently bind to the sialic acid residues in mucin. Our research group has previously developed cycA-loaded micelles containing 3-(acrylamido)phenylboronic acid for improved mucoadhesion and sustained release of cycA for up to 2 weeks.^13^

Of these mechanisms of mucoadhesion, NPs capable of forming covalent bonds with mucin should result in the greatest retention on the ocular surface. While thiomers have shown exceptional mucoadhesive properties and the potential for treatment of various diseases such as DED,^26^ there exist roadblocks to their use in formulations. Thiomers are unstable and prone to oxidization of their thiols at pH ≥ 5.^27^ Preactived thiomers represent a much more stable solution as the thiols moieties of the polymer are first reacted with another thiol-bearing small molecule forming disulfide bonds. These disulfides then undergo a disulfide exchange reaction in the presence of mucin, wherein the NP becomes bonded to the mucin and the thiol-bearing small molecule is released. This synthetic scheme was pioneered by the Bernkopf-Schnurch group, who utilized various therapeutic thiol-based leaving groups such as 2-mercaptonicotinic acid^28^ or N-acetyl cysteine (NAC).^29^

To increase micelle retention, incorporating a preactived thiomer within the copolymer backbone provides a means of both mucoadhesion and release of a therapeutic thiol bearing small molecule for treatment as a function of mucosal interaction. Herein, we describe the inclusion of an NAC-conjugated preactivated thiomer into the copolymer capable of self assembling into micelles for the release of cycA and treatment of DED. NAC is a commonly used mucolytic component in the treatment of DED and was therefore selected as the thiol leaving group.^30^ Although this formulation was developed for the treatment of DED, this material platform has the potential to be loaded with different drugs or conjugated with other thiol-bearing therapeutics for treating several diseases that affect mucosal surfaces throughout the body. Specifically, the conjugation of cysteamine and glutathione were also studied.

## Materials and methods

### Materials

All chemicals and solvents were purchased from Sigma-Aldrich (Oakville, Ontario) and used as obtained, unless otherwise specified. 3-(acrylamido)phenylboronic acid (98%; 3-AAPBA) was purchased from Sigma-Aldrich and purified by recrystallization in purified water prior to use. Azobis (isobutyronitrile) (AIBN) was purified by recrystallization in methanol (MeOH). Poly (D,L-lactide), 4-cyano-4-[(dodecylsulfanylthiocarbonyl)sulfanyl]pentonate with molecular weight 5 kDa (PLA-CDP) was purchased from Sigma-Aldrich. Purified water with a resistivity of 18.2 MΩ cm was prepared using a Milli-pore Barnstead water purification system (Graham, NC, USA). Phosphate buffered saline (PBS 10X, 1M, pH 7.4) was purchased from BioShop (Burlington, Ontario) and diluted 10x to obtain a 0.1 M (1x) solution with purified water prior to use. Monobasic sodium phosphate monohydrate (NaH_2_PO_4_ • H_2_O) and dibasic sodium phosphate heptahydrate (Na_2_HPO_4_ • 7H_2_O) were purchased from EMD Chemicals (Darmstadt, Germany) and used to make a 10 mM sodium phosphate buffer (Na-PB) in purified water. Regenerated cellulose dialysis membranes with a molecular weight cut off (MWCO) of either 3.5 or 6-8 kDa were purchased from Spectrum Laboratories Inc. (Rancho Dominguez, CA, USA). EZFlow® 13 mm high pressure liquid chromatography (HPLC)-grade nylon syringe filters with 0.45 and 0.2 μm pore sizes were purchased from Foxx Life Sciences (New Hampshire, USA).

### Pyridyl disulfide ethyl methacrylate synthesis

The synthesis of the monomer pyridyl disulfide ethyl methacrylate (PDSMA) involved an intermediate product, pyridyl disulfide alcohol (PDSOH) which was synthesized and purified according to a modified literature source.^31^ Aldrithiol-2 (1.250 g, 5.65 mmol; 1 eq.) was dissolved in 15.0 mL of MeOH and stirred at constant speed. Glacial acetic acid (2.5 mL, 43.70 mmol; 7.7 eq.) was added to the stirring solution dropwise using an addition funnel. A solution of β-mercaptoethanol (0.3 mL, 4.30 mmol; 0.72 eq.) in MeOH (10.0 mL) was prepared and added dropwise to the stirring solution using the addition funnel. The reaction was left stirring at room temperature for 24 h. Solvent volume was reduced using rotary evaporation and the crude product was redissolved in 25.0 mL of dichloromethane (DCM). Extractions were performed against saturated NaHCO_3_ (2 × 25 mL), purified water (1 × 25 mL) and saturated brine (360 g/L of NaCl in purified water; 1 × 25 mL). The organic phase was then dried with MgSO_4_, and gravity filtered. Solvent volume was reduced using rotary evaporation, resulting in final yellow oil product. Subsequently, silica column chromatography was used for purification (2:3 Hexane/Ethyl Acetate (EtOAc)) where PDSOH had an R_f_ ∼ 0.5 from thin layer chromatography (TLC) in these solvents. The collected samples of PDSOH were combined and the solvent volume reduced by rotary evaporation, to obtain the final product as a yellow oil.

PDSMA, synthesized by reaction of PDSOH with methacryloyl chloride, was prepared based on a modified literature protocol.^31^ PDSOH (0.564 g, 3.01 mmol; 1 eq.) was dissolved in anhydrous DCM (10.0 mL). *N,N*-Diisopropylethylamine (DIPEA) (1.05 mL, 6.02 mmol; 2 eq.) was added dropwise to the stirring solution, which was then placed in an ice bath to cool. A solution of methacryloyl chloride (0.38 mL, 3.91 mmol; 1.3 eq.) in anhydrous DCM (10.0 mL) was then added dropwise to the stirring reaction over 30 min by an addition funnel. The reaction was stirred at constant speed for 24 h and then allowed to warm to room temperature. The colour of the solution changed from yellow to dark brown overnight. The solution was then transferred to a separatory funnel and extractions were run against 1M HCl (1 × 20 mL), 1M NaOH (1 × 20 mL), PBS (1 × 20 mL), and a saturated brine wash (360 g/L of NaCl in purified water; 1 × 20 mL), followed by drying with MgSO_4_, gravity filtering, and solvent volume reduction by rotary evaporation. The crude product was a yellow oil which was purified by silica column chromatography (3:1 Hexane/EtOAc). The final product had an R_f_ ∼ 0.3 from TLC in these solvents. PDSMA column samples were collected, and the volume reduced with rotary evaporation to obtain final product as a yellow oil.

### Reversible addition-fragmentation chain-transfer (RAFT) polymer synthesis

Polymers produced were prepared using the same molar feed ratios as in previous work, with the only difference being an exchange of the mucoadhesive monomer component depending on the polymer to be made. Poly (D,L-lactide-*block*-(methacrylic acid*-co*-pyridyl disulfide ethyl methacrylate)) denoted LMS, and Poly (D,L-lactide-*block*-(methacrylic acid*-co*-3-(acrylamido)phenylboronic acid)) denoted LMP, were synthesized using reversible addition-fragmentation chain-transfer (RAFT) polymerization. The use of PLA for the hydrophobic segment stems from its history of being used in biomedical applications, including micelles, and its long-standing FDA approval.^32^ Methacrylic acid (MAA) was passed through a column packed with inhibitor remover beads. For synthesis of LMS and LMP, the method proposed by Prosperi-Porta et al. was used to make polymers with a feed ratio of 80:20:1.4:0.2 (MAA/(PDSMA or 3-AAPBA)/PLA-CDP/AIBN respectively).^13^ These polymers were designated LMS-20 and LMP-20 in accordance with previous terminology (20 mol % PDSMA or 3-AAPBA in the hydrophilic block).^13^ MAA (300 mg, 3.49 mmol), 3-AAPBA (166.4 mg, 0.87 mmol) or PDSMA (222.5 mg, 0.87 mmol), PLA-CDP (312 mg, 0.061 mmol), and AIBN (1.43 mg, 0.0087 mmol) were mixed with 7.8 mL of 9:1 dioxane/water (% v/v) to make a 10 % (w/v) solution in a 25 mL round-bottom flask. The reaction mixture was purged with nitrogen for 30 min, then heated in an oil bath to 70 °C for 24 h with constant stirring. The solvent volume was then reduced by rotary evaporation and the copolymers were redissolved in THF and isolated by precipitation into 10 times excess cold anhydrous diethyl ether (3 × 250 mL) with filtration through a Büchner funnel apparatus. The copolymer was left partially covered in the fume hood to dry for 48 h. The molecular weight and composition of the copolymers were determined by ^1^H NMR (Bruker AV 600 MHz) in deuterated dimethyl sulfoxide (DMSO-d_6_).

### Post-polymerization modification

To produce the final versions of the LMS-20 polymer, a post-polymerization modification was performed with small thiol molecules through a thiol/disulfide exchange reaction, based on a protocol modified from the one proposed by Peng et al.^33^ In a typical reaction procedure, 30 mg of LMS-20 polymer (20.6 kDa, 1.69 × 10^−3^ mmol) was dissolved in 6 mL of acetone to make a 5 mg/mL solution. A small amount of glacial acetic acid (∼3 drops) was added to ensure an acidic environment to improve reaction kinetics and prevent thiol oxidation. The small thiol molecule of choice; either Cysteamine (Cys), Glutathione (GSH), or *N*-acetyl cysteine (NAC), was dissolved separately in a suitable solvent (acetone for NAC; purified water for Cys & GSH) to a concentration of 50 mg/mL. From this stock solution, a volume was transferred to the polymer solution such that the molar ratio of thiol to pyridyl group was 3:2 (i.e., 5.4 mg or 0.0696 mmol of Cys, 21.4 mg or 0.0696 mmol of GSH, 11.36 mg or 0.0696 mmol of NAC). The reaction was left to stir at room temperature overnight. Each reaction mixture was then transferred to Spectra/Por® 3.5 kDa MWCO regenerated cellulose dialysis tube and dialyzed against purified water for 4 days, with periodic changes of the water. Subsequently, dialyzed polymer was transferred to 20 mL glass vials, frozen, and lyophilized over 2 days to obtain the final product, a yellow powder. The composition of the modified copolymers was determined by ^1^H NMR (Bruker AV 600 MHz) in DMSO-d_6_ and/or deuterated water (D_2_O). The final copolymer was named LMA-20 for NAC-modified LMS-20.

### Polymer nanoprecipitation

To form nano-micelles out of the synthesized polymers, the polymer of choice was dissolved in acetone at a concentration of 20 mg/mL, then heated in an oven to 50 °C for 5 min. To this polymer solution, 3-5 drops of Na-PB were added and the mixture reheated in the oven at 50 °C. The process was repeated until it resulted in a clear solution.

The polymer solution was then added dropwise (∼1 drop/s) through an 18G needle into a stirring solution of Na-PB. The mixture was covered with an aluminum tent to allow acetone evaporation and left stirring at 850 RPM for 48 h. The final concentration of nano-micelles was 10 mg/mL, and the final pH was adjusted to 7.4. Prior to use, the micelles were filtered through 0.45 µm pore nylon syringe filters and stored in the fridge at 4 °C.

Drug-loaded micelles were prepared in a similar manner. CycA was dissolved in acetone to a concentration of 5 mg/mL and from this solution the desired amount of drug was transferred to the polymer solution described above. Subsequently, the polymer/drug mixture was added dropwise to PBS (drug release, rheological studies, and viability studies), in the same manner.

### Micelle size determination

The particle size of LMA-20 and LMP-20 nano-micelles was determined using dynamic light scattering (DLS) using a Brookhaven 90 Plus Particle Size Analyzer to get the average effective diameter and polydispersity index (PDI) of the nano-micelles. 2 mL of nano-micelle solution in PBS at a concentration of 1 mg/mL and pH 7.4 was added to a polystyrene two transparent sided cuvette; a concentration chosen to obtain an appropriate count rate.

### Micelle morphology determination

The shape and structure of the LMA-20 and LMP-20 nano-micelles was observed by Transmission Electron Microscopy (TEM) (Jeol TEM-1200EX transmission electron microscope with an 80 kV electron beam). TEM samples were prepared by air-drying 2 µL of 1 mg/mL micelle solution in Na-PBS on a carbon coated 400 mesh copper grids prior to analysis. Samples were measured at a level of magnification of 50 000x.

### Critical micelle concentration

The Critical Micelle Concentration (CMC) was determined through the pyrene fluorescence method.^34^ A stock solution of pyrene in acetone (100 µg/mL) was prepared and 4 µL transferred to 4 mL glass vials, allowing the acetone to evaporate overnight to form a pyrene film. Solutions of LMA-20 micelles in PBS were prepared in a range of concentrations (10^−6^ mg/mL to 1 mg/mL) by dilution from a 10 mg/mL stock solution. 1 mL of each concentration was added to each glass vial containing pyrene. Mixtures were shaken for 24 h, then analyzed using a BioTek Cytation 5 Cell Imaging Multi-Mode Reader in fluorescence intensity mode (Vermont, USA) with an excitation wavelength of 340 nm, emission wavelengths of 373 and 383 nm, and with a bandwidth of 9 nm. To determine the CMC, the fluorescence intensity ratio (I_373_/I_383_) as a function of the logarithmic polymer concentration was plotted.

### Determination of mucoadhesion by zeta potential

LMP-20 and LMA-20 micelle samples were prepared as described. A stock solution of porcine stomach mucin (PSM) Type III (Sigma Aldrich) at 10 mg/mL in PBS was prepared and left stirring for 24 h. Control samples of micelle and PSM were diluted to 5 mg/mL prior to incubation and analysis. A 1:1 mixture (v/v) of 10 mg/mL micelle solution and 10 mg/mL PSM was prepared to a final concentration of 5 mg/mL. Samples were placed in an incubating shaker at 37 °C for 1.5 h to allow for particle interactions prior to analysis. Samples were then placed into polystyrene two-sided cuvettes, and an AQ 1204 probe was placed into the cuvette. Samples were analyzed using the zeta-potential function on the Brookhaven 90 Plus Particle Size Analyzer at 25 °C.

### Determination of mucoadhesion by rheology

Mucoadhesive properties of LMA-20 and LMP-20 were further compared through rheological measurements on a TA Instruments (Delaware, USA) discovery hybrid rheometer (DHR; Discovery HR-20), equipped with a C20/1° cone and Peltier plate combination, running TRIOS software. Measurements of complex viscosity were made at angular frequencies from 0.1 to 100 s^−1^ and a strain of 1% at a constant 15 °C.

Samples of LMA-20 and LMP-20 micelles at 10 mg/mL in PBS were made in triplicate. A 50 mg/mL mucin stock solution was prepared in PBS. Mixtures of micelle and mucin were prepared by mixing micelle and mucin solutions 1:1 (v/v) and were incubated at 37 °C overnight in an incubating shaker, while controls of micelles and mucin were prepared by mixing 1:1 (v/v) with PBS.

Rheological synergism was calculated to determine the extent of interaction between mucin and micelles through the following equation (1):

(1) $$\Delta G^{\prime }=G_{mix}^{\prime }-\left(G_{micelles}^{\prime }+G_{mucin}^{\prime }\right)$$

where $G_{mix}^{\prime }$ is the storage modulus (mPa) of the micelle/mucin mixture, $G_{micelles}^{\prime }$ is the storage modulus (mPa) of the micelle control, and $G_{mucin}^{\prime }$ is the storage modulus (mPa) of the mucin control, all at an intermediate angular frequency of 10 s^−1^.^35^

### Drug entrapment efficiency (EE) and drug loading (DL)

CycA loaded LMA-20 nano-micelles were prepared as described. LMA-20 nano-micelle samples at a polymer concentration of 10 mg/mL and cycA concentration of 1.5 mg/mL were obtained in PBS. A small sample of each was taken for encapsulation efficiency (EE) and drug loading (DL) studies. Briefly, micelle samples were transferred to an Eppendorf tube and centrifuged at 5000 RPM for 30 min. The supernatant was then diluted 100x in a separate Eppendorf tube with acetonitrile (ACN), representing the entrapped drug sample, as the NPs are stable in solution and allow for the drug to remain within the aqueous supernatant. The precipitate was mixed with 1 mL of ACN to make up the free drug solution, as the hydrophobic drug that is not encapsulated in the NPs will precipitate from the aqueous solution, allowing purification via centrifugation. The free drug solution was then diluted 100x in a separate Eppendorf tube with fresh ACN, representing the free drug sample. Samples were then filtered through 0.2 µm pore nylon syringe filters, transferred to 200 µL disposable inserts in 1 mL HPLC vials and run on an Agilent 1260 Infinity II HPLC utilizing a binary HPLC pump, autosampler, UV/Visible detector set to a wavelength of 210 nm, with an ACN/Water (80:20 v/v) mobile phase flowing at 0.7 mL/min through a Phenomenex C18 (150 × 4.6 mm, 5 µm particle size) column. Column temperature was set at 60 °C, and injection volume to 20 µL. The concentration of the samples was determined by generating a standard calibration curve of cycA in the mobile phase.

### CycA release profile

Remaining cycA loaded LMA-20 micelle samples from the EE/DL studies were used for the release studies. 250 µL of each micelle sample was transferred to Spectra/Por® 6-8 kDa MWCO regenerated cellulose dialysis tubing and placed in ∼7 x sink conditions of PBS, i.e., 100 mL for 1.5 mg/mL cycA loaded micelles, at 37 °C in an incubating shaker (100 RPM). Release samples of 10 mL were taken at intervals of 1, 3, and 10 h, as well as 1, 2, 3, 7, and 10 days. Collected samples were frozen and lyophilized, then reconstituted in ACN/deionozed water (80:20, v/v), concentrated five-fold. Samples were then shaken for 1 h to phase separate the salt and organic phase and extract the drug into the ACN. The organic phase of samples was filtered through 0.2 µm pore nylon syringe filters into Eppendorf tubes and stored at 4 °C until further use. Release samples were transferred to 200 µL disposable inserts in 1 mL HPLC vials and run on HPLC as described above using the same instrument and methods.

### *In vitro* cellular viability

The cellular response to blank LMA micelles was determined following incubation with human corneal epithelial cells (HCECs) by MTT assay and Live-Dead imaging. LMA-20 nano-micelles were prepared as previously described. The micelles were then incubated in a closed, transparent glass vial at 25 °C overnight under UV light to ensure sterility prior to testing. For both MTT assay and Live-Dead imaging, 20 000 HCECs in 200 µL of media were seeded per well of a 96 well plate. After 24 h, this media was removed and replaced with 180 µL of fresh media. Both negative and positive control wells then received 20 µL of sterile PBS. For LMA testing, the wells were treated with 20 µL of sterile PBS containing a given concentration of LMA-20 micelles which would result in a final concentration of 500 µg/mL, 250 µg/mL, and 50 µg/mL of micelles (n = 4 per concentration).^13,36,37^ The cells were then incubated with the micelles for 24 and 48 h before assessment. To produce positive controls, just prior to MTT or Live-Dead testing, the media was removed, and the cells were treated with 100 µL 0.25% (v/v) Triton X-100 for 3 min to ensure that the cells did respond to a cytotoxic substance. The cells were then washed three times with 200 µL of sterile PBS. Negative controls were untreated.

For MTT assay, 3-(4,5-dimethylthiazol-2-yl)−2,5-diphenyltetrazolium bromide (MTT) powder was dissolved in sterile PBS to a concentration of 5 mg/mL. The working solution was then dissolved to 10% (v/v) in media. After 24 or 48 h, the cell media was removed and replaced with 100 µL of the diluted MTT media. The plates were incubated for 3 h before the supernatant was removed and the formazan crystals produced were dissolved in 200 µL of DMSO for 15 min. Finally, the absorbance was quantified by a SpectraMax® ABS Plus UV–vis micro-plate reader (Molecular Devices, San Jose, California, USA) at a wavelength of 570 nm. Cellular viability was determined by comparing the absorbance of treated wells (ABS_treated_) to the negative control absorbance (ABS_control_) using equation (2).

(2) $$Viability\;\left(\%\right)=\left(\frac{{ABS}_{Treated}}{{ABS}_{Control}}\right)\times 100$$

For Live-Dead imaging, after 24 or 48 h, all the cells were stained with a calcein-AM/ethidium homodimer-1 fluorescence kit (Thermo Fisher Scientific) and visualized with an Olympus IX51 inverted fluorescent microscope (Shinjuku, Tokyo, Japan).

### Statistical analysis

Error bars represent the standard deviation. To test for significance in the results, Minitab 18 was used to run a one-way analysis-of-variance (ANOVA), with a post-hoc Tukey’s test, or Welch’s test to account for unequal variances, to obtain *p*-values. The level *p* < 0.05 was set for significance, *p* < 0.01 for very significant, *p* < 0.001 for highly significant.

## Results and discussion

### Monomer synthesis

**Figure 1.**
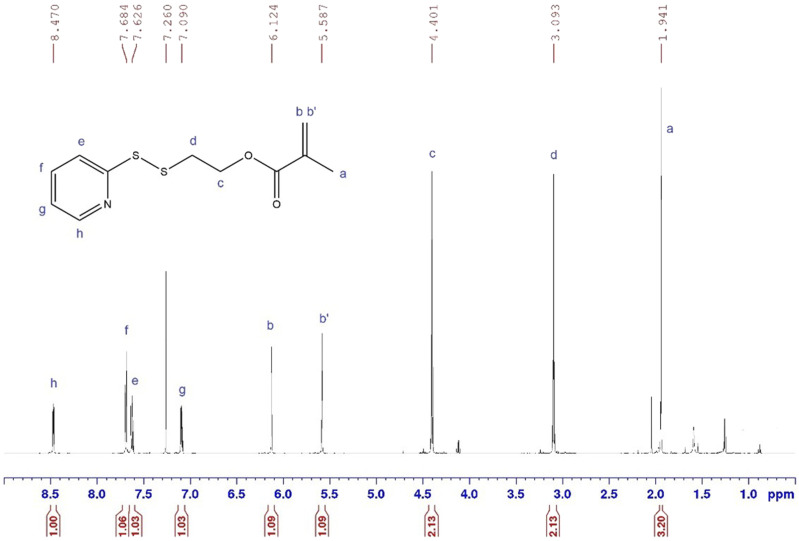
^1^H NMR spectrum of purified PDSMA product in CDCl3. δ [ppm] = 6.1, 5.9 attributed to the introduction of the acrylate group and δ [ppm] = 1.95 indicative of the methyl group correspond to successful monomer synthesis.

The monomer PDSMA was synthesized in this study to facilitate subsequent modification via a thiol/disulfide exchange reaction.^38^ The ^1^H NMR analysis of the final PDSMA monomer is shown in Figure 1. The reaction yields from the intermediate PDSOH and final PDSMA synthesis were 52.7% and 49.5% respectively. Previously, a 72% yield was reported, which is significantly higher than what was achieved in this work.^31^ However, optimization of the reaction conditions as well as extraction protocols and purification may lead to improved yields.

### Copolymer synthesis

The synthesis of amphiphilic block copolymers using the RAFT polymerization technique has been used extensively in the literature.^39^ Based on previous work in the Sheardown Lab, the polymers LMP-20 and LMS-20 were successfully synthesized using the RAFT polymerization technique.^13^ The desire was to synthesize both polymers to investigate the mucoadhesive properties imparted by the PBA and preactivated thiomer moieties. The chemical composition and molecular weight were characterized using ^1^H NMR. Successful polymer synthesis was characterized by an elimination of acrylate peaks at δ ∼ 6.0 – 5.5 and introduction of methyl and ethyl peaks from the polymer backbone at δ ∼ 2.0 – 0.5.

The chemical composition of the polymers was determined through proton integration of the ^1^H NMR spectra. Commercially available PLA-CDP was analyzed by GPC and found to have a number average molecular weight (M_n_) of 5115 g/mol, weight average molecular weight (M_w_) of 5597 g/mol, and a PDI of 1.09. With the molecular weight of the lactic acid repeat unit being 72.06 Da, and the non-repeat unit portion amounting to a molecular weight of 388.4 Da, using the M_n_ from GPC, there are 65.6 repeat units per polymer chain. By integrating the NMR spectra for the individual proton of PLA at δ = 5.2 to 65.6, it was possible to calculate and normalize the final monomer molar ratio and subsequently the molecular weight of the polymer. The results for both polymers are shown in Table 1.

**Table 1.** Polymerization data for LMP-20 and LMS-20 obtained from analysis of ^1^H NMR chemical shift.

Polymer	Molecular Weight (g/mol)	Molar Feed Ratio (LA:MAA:3-AAPBA or PDSMA)	Final Molar Ratio (LA:MAA:3-AAPBA or PDSMA)	Ratio of MAA:3-AAPBA or PDSMA
LMP-20	19,932	50:40:10	32.3:53.7:14.0	3.8:1
LMS-20	20,556	50:40:10	33.6:54.5:11.9	4.6:1

The results in Table 1 show similar chemical composition and molecular weight between the two polymers. The final molecular weight of the polymers is ∼20 kDa, which is an acceptable polymer MW for renal clearance (MWCO ∼70 kDa),^40^ an important property to ensure elimination of the material from the body. Properties of the LMP-20 polymer also agree with previous reported results.^13^ The results show a higher amount of 3-AAPBA in LMP-20 compared to the amount of PDSMA in LMS-20, a difference amounting to a 23% increase in the number of 3-AAPBA groups (28 repeat units from ^1^H NMR) compared to PDSMA groups (23 repeat units from ^1^H NMR) per polymer. This result must be considered when analyzing results from mucoadhesive studies as these are the major mucoadhesive components of the polymers.

Due to issues encountered in the solubility of both polymers, because of their amphiphilic nature, ^1^H NMR was used to estimate the MW of each polymer. The limitations on accuracy of MW determination from ^1^H NMR suggest that future work should investigate a more robust method such as GPC to determine both the MW and the PDI of these polymers.

### Post polymerization modification

**Figure 2.**
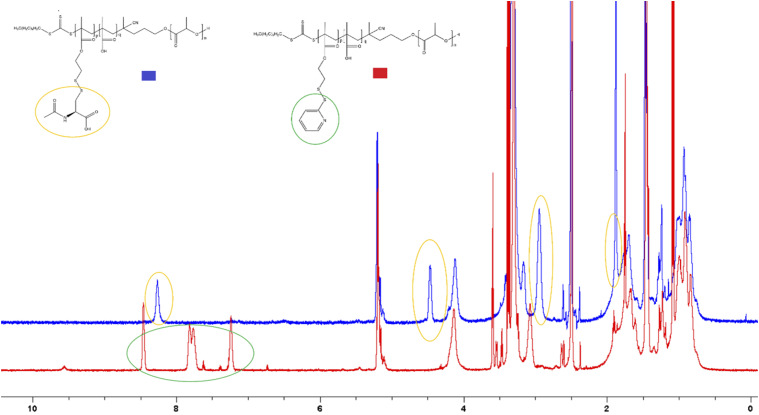
^1^H NMR spectra comparison between LMS-20 (red) and LMA-20 (blue). Yellow circles represent the new peaks attributable to the successful modification with NAC; green circles represent the peaks of the PDS group that can be seen to have disappeared following successful modification.

Modifications to the LMS-20 polymer were required as the 2-pyridinethione leaving group can be cytotoxic.^28^ Based on the literature suggesting that small, less reactive thiol molecules such as NAC can provide enhanced mucoadhesion in comparison to more reactive thiol ligands such as pyridine groups, the choice was made to modify LMS-20 with NAC.^29^ NAC is also commercially available as a 5% w/v ophthalmic solution (Ilube® by Rayner) to treat DED. Modifications were made through a thiol/disulfide exchange reaction and results for the modification with NAC can be seen in the ^1^H NMR spectrum comparison shown in Figure 2. The successful incorporation of NAC into the polymer resulted in elimination of PDS peaks between δ ∼ 7.0 – 8.5 and the introduction of novel NAC peaks at δ ∼ 8.3, 4.5, 3.2 and 1.9. Successful modifications were also performed with cysteamines and glutathione, however, difficulties with the nanoprecipitation method made these polymers not viable for further exploration. Future work should investigate the solubility of these modified polymers in different solvents to allow for the use of nanoprecipitation to form micelles.

### Micelle size and morphology

The effective diameter and PDI of the micelles were determined by DLS (Table 2). A comparison between the LMP-20 and LMA-20 micelles reveals a significant difference (Tukey; *p* < 0.001) in their sizes. A significant increase in micelle size after cycA loading was also observed (Tukey; *p* < 0.01). Similar results for increased size after drug loading have been shown in the literature with loading of dexamethasone into micelles,^24^ as well as previous results with cycA loaded LMP-20 micelles (data unpublished). The PDI values for nanoparticles vary widely from fairly monodisperse distributions (*p* < 0.05) to large distributions (>0.7).^12^ The PDI of the LMA-20 and LMP-20 micelles is around 0.3, representing a wider distribution. These results are comparable to those obtained from other polymeric micelle formulations.^13,41,42^

**Table 2.** LMP-20 and LMA-20 micelle size from DLS measurements. PDI values represent the mean (n = 3). Effective diameter values represent the mean ± standard deviation (SD) (n = 3).

	Empty micelles		CycA loaded micelles	
Polymer	Effective diameter (nm ± SD)	PDI	Effective diameter (nm ± SD)	PDI
LMP-20	72 ± 3	0.30	-	-
LMA-20	64 ± 5	0.28	71 ± 5	0.32

**Figure 3.**
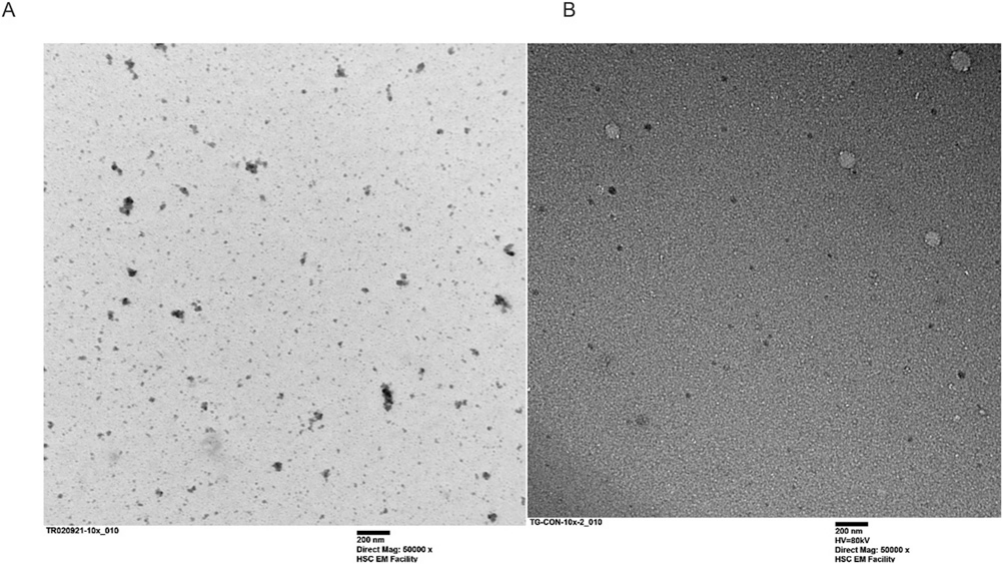
Transmission Electron Micrograph of the LMA-20 (left panel) and LMP-20 (right panel) micelles with distinct spherical morphology.

TEM of the micelles (Figure 3) show spherical morphology for both LMP-20 and LMA-20 micelles, with approximate size from the TEM images agreeing with the DLS results.

### Determination of critical micelle concentration

**Figure 4.**
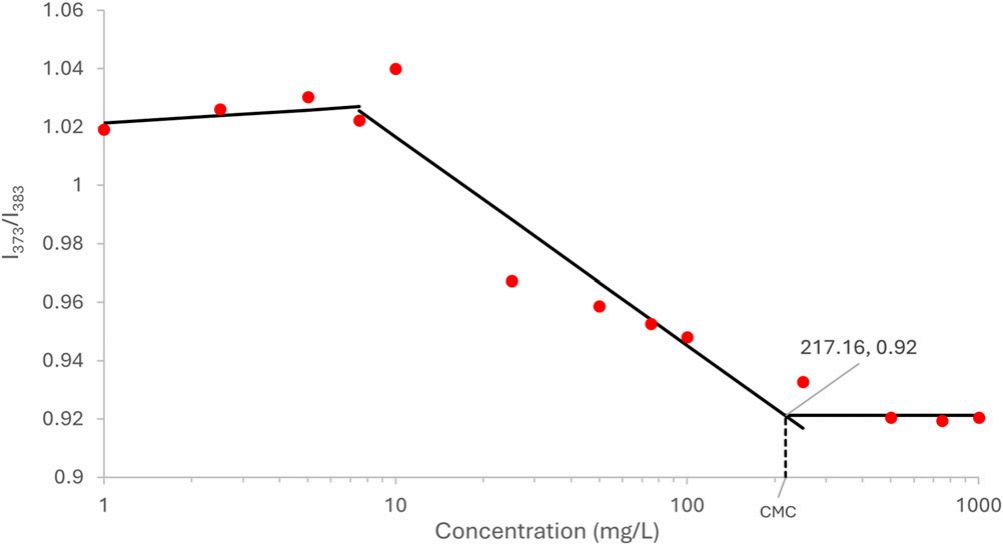
CMC as determined by the pyrene fluorescence intensity ratio at 373 nm and 383 nm, with excitation of 340 nm, measured at different concentrations of LMA-20 polymer in PBS. Concentration is plotted on a logarithmic scale. Each value represents the result from a single measurement (n = 1).

The CMC is an important parameter of a micellar drug delivery system as it provides insight into the stability of system. A CMC lower than the concentration of intended application is desirable as this ensures that the micelle will stay intact and not disassemble upon application. To measure the CMC, the pyrene fluorescence method was used in which different concentrations of LMA-20 micelle solution were incubated with a fixed concentration of pyrene. As the concentration of LMA-20 increases, the pyrene will preferentially partition into the hydrophobic core of the micelles and the ratio of the intensity of the emitted light at λ = 373 nm and 383 nm will rapidly change (I_373_/I_383_).^43^ This result can be seen in Figure 4, where the CMC is determined as the intersection of the best fit line to the variable region with the nearly horizontal region at high polymer concentration.^34^ A CMC of 217 mg/L was obtained for the LMA-20 polymer. For all practical applications of this formulation, such a low value for the CMC is considered acceptable. This is a highly conservative estimate as some in literature use the inflection point of the highly variable region, or the intersection of the highly variable region with the nearly horizontal region at low polymer concentration as the CMC, which would result in much lower values of the CMC.^43,44^

### Determination of mucoadhesion by zeta potential

**Figure 5.**
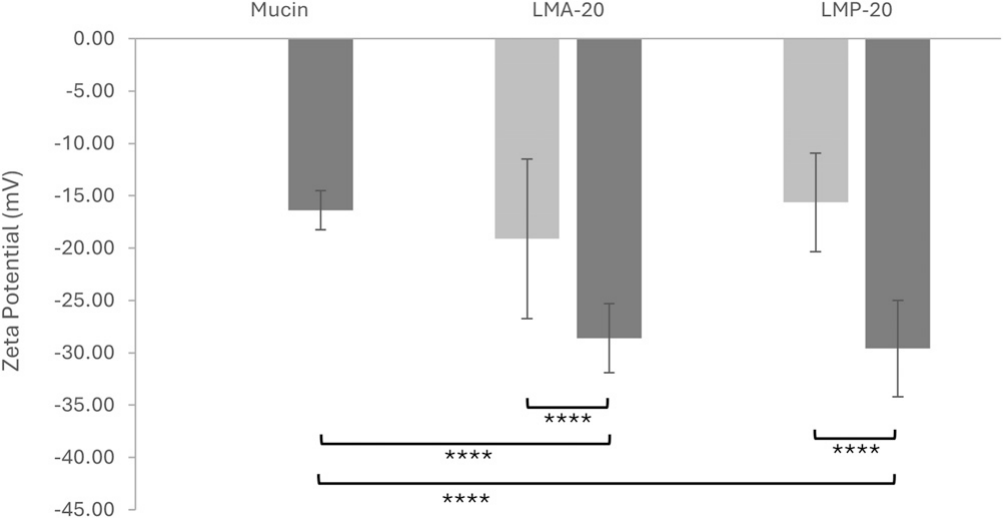
Zeta potential values for a mucin solution, LMA-20 sample, and LMP-20 sample, with (dark grey) and without (light grey) mucin. Each value represents the mean ± SD of 18 measurements (n = 3 samples). Welch’s test; *p* < 0.001 (****). All samples prepared in Na-PB.

To determine whether the micelles were mucoadhesive, zeta potential studies were performed (Figure 5). Control samples of mucin, LMP-20 micelles, and LMA-20 micelles were run along with micelle/mucin mixtures. Control samples had a negative zeta potential. The negative charge of the mucin can be attributed to sialic acid and sulfate residues, while that of the micelles can be attributed to carboxylic acid groups in the hydrophilic shell of the micelles.

The assumption is that a micelle/mucin mixture whose zeta potential is the same as either of the controls or that lies at an average value between the two controls, is not mucoadhesive. This is because it is not possible to determine whether the change in zeta-potential was simply due to the addition of micelles to the mucin, creating an average value of the zeta potential between the two species in solution. It was assumed that a significantly lower observed zeta potential between the micelle/mucin mixtures and their respective controls is due to mucin-particle interactions and not simply due to the mixture of the two.^45^ As was observed in Figure 5, a highly significant decrease in the zeta potential was seen upon mixing the LMP-20 and LMA-20 micelles with mucin, with respect to their controls and the mucin control (Welch; *p* < 0.001). This interaction between mucin and NPs is typically attributed in the literature to the adsorption of mucin to the particle surface, coating the particle and resulting in a change in the surface charge of the particles.^46^ Therefore, both LMP-20 and LMA-20 are predicted to be mucoadhesive based on this technique.

### Determination of mucoadhesion by rheology

To complement the zeta-potential study, a rheological investigation was performed to investigate mucoadhesive potential of the LMA-20 and LMP-20 micelles. A theoretical value of the complex viscosity for the micelle/mucin mixture can be obtained by summing the complex viscosity of the micelle and mucin controls and comparing this to the experimental value obtained for the mixture. If the calculation for the rheological synergism results in a positive value that is significantly different from zero, then it is predicted that an interaction occurred between mucin and the micelle.^47^

**Figure 6.**
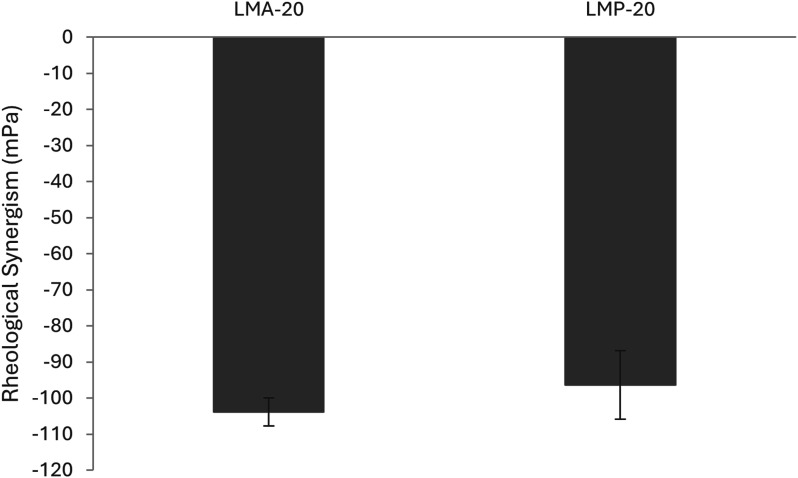
Rheological synergism as calculated from storage modulus for LMA-20 and LMP-20 experiments at an angular frequency of 10 s^−1^. Values represent the mean ± SD (n = 3). No statistical difference observed between the two micelle formulations (Tukey; *p* > 0.05).

An oscillatory strain sweep was performed on all sample types to determine a strain in the linear viscoelastic region among all samples. To this end, a strain of 1% was chosen for the subsequent oscillatory frequency sweeps. The results were used to calculate the rheological synergism, which is reported in Figure 6 at an angular frequency of 10 s^−1^. The rheological synergism in this study had a highly significant difference from zero (Tukey; *p* < 0.001) and was negative for both LMA-20 and LMP-20 mixtures. This result is not unexpected as many have reported similar results for NP formulations, as well as some polymer formulations.^47–50^ As was reported by Hägerström and Edsman, it is possible that negative values of rheological synergism can result from an interaction between mucin and polymer, resulting in weaker, rather than stronger, properties of the mixture in comparison to the controls.^51^ Eshel-Green and Bianco-Peled found similar negative synergism with their acrylated poloxamer micelles.^48^ Their explanation for the phenomenon is that commercial mucin contains large aggregates which the micelles may be small enough to penetrate. Once the micelles penetrate these large aggregates, they separate the mucin glycoprotein chains from one another and adsorb individual mucin strands to their surface, resulting in degradation of the mucin aggregates, and under shear, reduction in viscosity.^48^ Further, given that previous work using SPR demonstrated interactions between the LMP-20 materials and mucin, it can be concluded that there are mucin interactions. However, further investigation is necessary to provide quantification of the adhesion. Therefore, these rheological studies provide evidence of mucoadhesion for either micelle formulation, though based on the limitations of this technique,^51^ future studies either *ex vivo* or *in vivo* would be beneficial in comparing the two formulations.

### CycA loading and release

Several relevant ocular therapeutics have been encapsulated effectively into the LMP-20 formulation in past work. However, unpublished results suggest that the PBA group is thought to hinder the encapsulation of certain compounds. Therefore, in addition to providing a different method of binding to the mucin, altering the binding moiety may allow for the encapsulation of compounds that were potentially sub-optimal with the LMP-20 formulation.

CycA is an immunosuppressant drug employed in the treatment of DED through the instillation of eye drops.^52^ Many have been developing mucoadhesive formulations to compete with the market staple Restasis® (0.05 % w/w cycA ophthalmic emulsion).^13,53,54^ The Sheardown Lab has previously shown the ability to encapsulate cycA using the LMP-20 formulation.^13^ Therefore, cycA represented an appropriate starting point.

Prior to performing a drug release, the EE and DL content of the LMA-20 micelle formulation were determined; the results are reported in Table 3. The LMA-20 micelles had an EE of 90.6 ± 7.4%, forming a 13.8% (w/w) cycA formulation. Prosperi-Porta et al. reportedly obtained a DL of 15% (w/w) for the LMP-20 micelles loaded with cycA.^13^ Unpublished results have shown similar DL of 13%–15% (w/w) for LMP-20 batches of similar composition to those produced herein. In comparison to the market leader for DED treatment Restasis®, the LMA-20 micelles entrap ∼ 3x the amount of cycA at a similar concentration.

**Table 3.** Drug EE and DL of LMA-20 polymeric micelles with cycA. All numbers represent the mean ± SD (n = 3).

	CycA Feed (mg/mL;±SD)	Entrapped CycA (mg/mL;±SD)	EE (%;±SD)	DL (% w/w;±SD)
LMA-20	1.76 ± 0.28	1.61 ± 0.37	90.6 ± 7.4	13.8 ± 2.8

**Figure 7.**
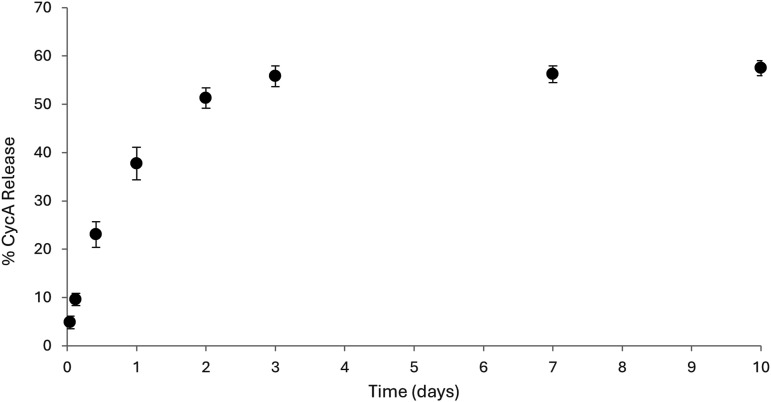
Cumulative release profile of CycA from LMA-20 micelle formulation (0.16% CycA formulation) in PBS. Each value represents the mean ± SD (n = 3).

Release of cycA from the LMA-20 micelles was studied to understand the potential use of the formulation for delivery of ocular therapeutics. As shown in Figure 7, sustained release for up to 3 days was observed. The LMA-20 micelles were able to release 57.5% (231 µg) of the entrapped drug after 10 days. It is possible that further release from the LMA-20 micelles could be expected after 10 days, although it is likely that the micelles would be cleared from the eye by this time and therefore additional sampling is unlikely warranted. While sustained release is the goal of the study, patient compliance is an important factor to consider. Habitual daily instillation is preferable to skipping days for patients, as routine is imperative for positive outcomes. Using a controlled release mechanism for drug release, the benefit of a less intense burst release coupled with flexibility for missed doses allows for human error to be minimized. While extended release is an attractive feature, release beyond 2 days is likely to be reinforced with an additional dose of the drug system.

### Cellular viability

**Figure 8.**
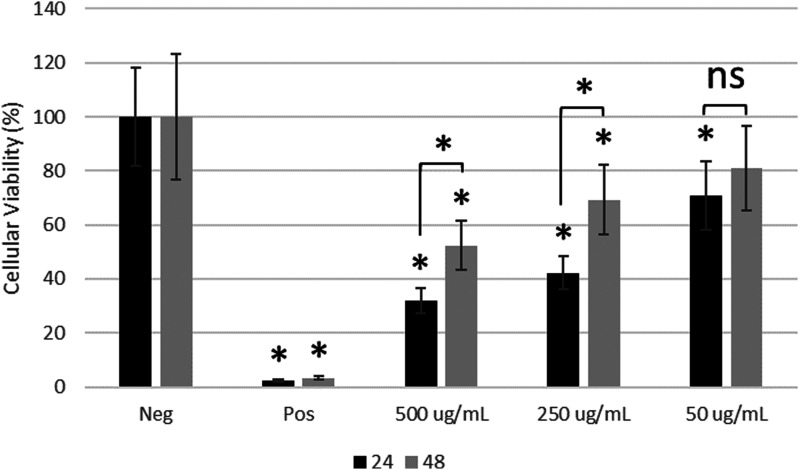
MTT metabolic assay of HCECs treated with LMA micelles at various concentrations. HCECs treated with 500 and 250 µg/mL of LMA micelles were statistically lower (*p* < 0.05) cellular viability compared to the untreated controls. After 24 h the HCECs treated with 50 µg/mL had a statistically lower (*p* < 0.05) viability compared to untreated controls but after 48 h the viability was not statistically different (*p* > 0.05). Over time the viability of HCECs treated with 500 and 250 µg/mL of LMA micelles statistically increased (*p* < 0.05). Data presented as mean ± SD (n = 4), significance determined by single factor ANOVA analysis followed by Tukey’s post-hoc analysis. ‘∗’ indicates significant difference (*p* < 0.05).

Following treatment with blank LMA micelles for 24 and 48 h, the results of the MTT assay are shown in Figure 8. All the wells treated with LMA micelles has a statistically lower cellular activity (*p* < 0.05) versus the untreated control except for HCECs treated with 50 µg/mL LMA micelles at 48 h. The result suggests that the LMA micelles do lower cellular metabolic activity, however, at the 24 h time point, HCECs treated with 50 µg/mL LMA micelles had a statistically higher (*p* < 0.05) cellular viability compared to HCECs treated with 500 or 250 µg/mL LMA micelles suggesting that the cytotoxic effect is dose-dependent. Furthermore, between 24 and 48 h the cellular viability for HCECs treated with 500 or 250 µg/mL LMA micelles statistically increased (*p* < 0.05) indicating that the cells recover following initial dosing. The positive control demonstrated that treatment with a cytotoxic agent does lead to cell death, as expected. Due to the previously determined safety of cycA, in Restasis® eye drop formulation that is currently on the market, it was determined that the materials would be tested for the viability of the delivery vehicle, allowing the potential for the vehicle to be used with a library of anterior ophthalmic drugs in the future. There is the potential for interactions between the vehicle and the drug; *in vivo* validation in a dry eye using our previous formulation demonstrated that these polymers do not interact adversely with the drug; the work will be the subject of a future manuscript. Furthermore, while the materials are capable of loading an increased concentration of cycA, the slow release and the drainage properties of the tears mean that it is unlikely that concentrations will reach cytotoxic levels. However, *in vivo* validation is a better measure of this.

**Figure 9.**
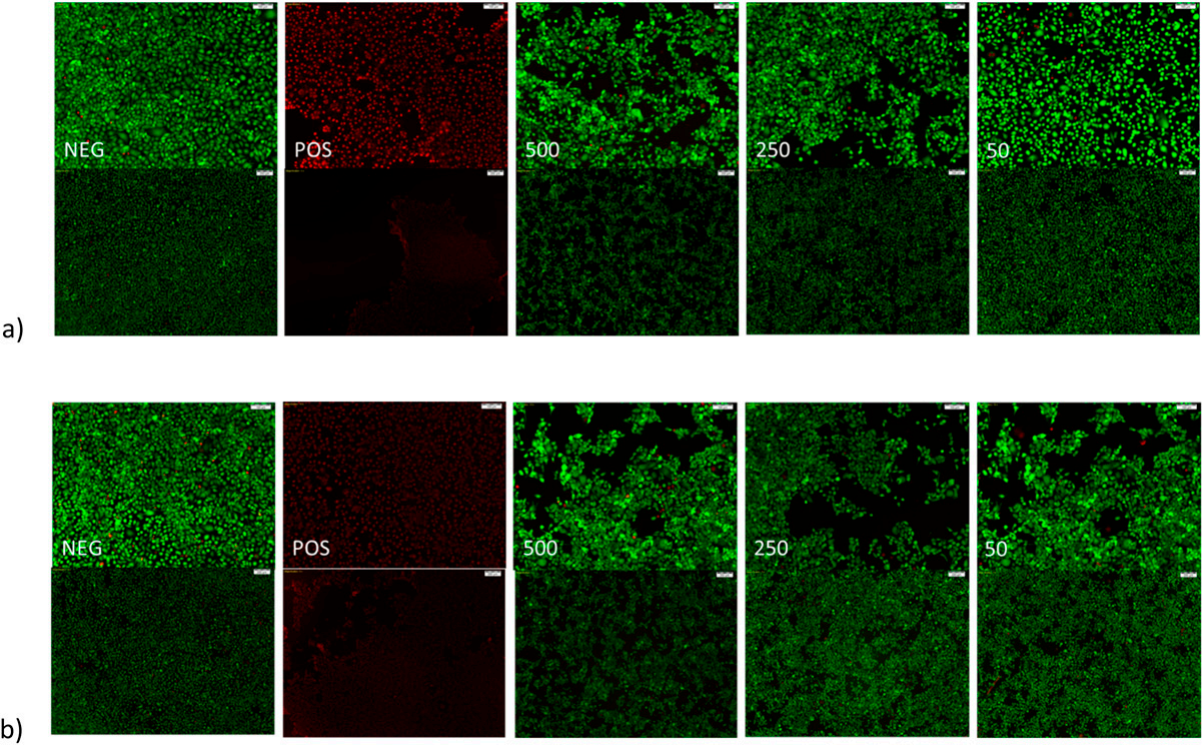
Live-Dead staining of HCECs following treatment with various concentrations of LMA micelles for (a) 24 h and (b) 48 h. Images show from left to right negative control (NEG), positive control (POS), 500, 250, and 50 µg/mL LMA micelles. Top images taken as 10× magnification (scale bars 100 µm) and bottom images at 4× magnification (scale bars 200 µm). Greater degree of cell lifting noted after treating with higher concentrations of LMA micelles.

The Live-Dead staining and subsequent imagining of HCECs treated with varying concentrations of LMA micelles are shown in Figure 9. Figure 9(a)) displays the stained cells after 24 h at 10× (top) and 4× (bottom) objective magnification while Figure 9(b)) displays the stained cells after 48 h at 10× (top) and 4× (bottom) objective magnification. From Figure 9 it can be observed that HCECs treated with higher concentrations of LMA micelles result in a lower area of cellular growth. This suggests that the LMA micelles may disrupt cell adhesion although additional studies are necessary to confirm that this is the case.

Overall, it can be concluded that LMA micelles may have a mild, dose dependent cytotoxic effect, potentially related to the presence of the thiol group. However, results suggest that the cells begin to recover following initial treatment. For the LMA micelles a CMC of 217 mg/L was determined. Therefore, treating HCECs with a LMA concentration above this value results in micelle formation while below this value the LMA polymer is in solution. This may explain the cytotoxic effect observed with 500 and 250 µg/mL of LMA micelles as it may in fact be that the acetone core is impacting the cells. Further drying of the micelles may improve this result. In the literature, micelles are often tested at a concentration of range of 1–500 µg/mL.^36,37,55–57^ Particularly, Sun et al. demonstrated that HCECs treated with 400 µg/mL of their micelle formulation resulted in lower cellular viability compared to lower micelle concentrations.^56^ Future work will focus on testing the developed LMA micelles in an *in vivo* model to better assess safety.

## Conclusions

Preactivated thiomer-based micelles with the potential to improve mucoadhesion, and provide sustained release of relevant ocular therapeutics, were successfully synthesized. A preactivated thiol monomer was synthesized (PDSMA) as confirmed by ^1^H NMR. Incorporation of this monomer in the synthesis of the amphiphilic block copolymers LMS-20 was confirmed by ^1^H NMR. Comparing to a previous amphiphilic block copolymer micelle formulation containing 3-AAPBA (LMP-20), the mucoadhesive properties imparted by the PBA and preactivated thiomer of LMS-20 were investigated. Modification of LMS-20 with NAC was done, and successful synthesis was confirmed with ^1^H NMR by the loss of aromatic peaks associated with 2-pyridinethione and incorporation of peaks associated with the NAC molecule. LMA-20 was the focus of this work as it contained the most relevant thiol modification for ocular applications and was capable of nanoprecipitation to form aqueous micelles with known methods. Spherical, micelles of LMA-20 and LMP-20 were formed with effective diameters of 64 ± 5 nm and 72 ± 3 nm and a critical micelle concentration of 217 mg/L. Both LMA-20 and LMP-20 were suggested to be mucoadhesive based on zeta-potential studies and rheological studies. LMA-20 micelles were able to entrap 3x the amount of cycA as the market leader Restasis® and release of cycA from the micelles was sustained for approximately 3 days. Testing with HCECs demonstrated that the LMA micelles had a dose dependent cytotoxic effect, but the cells recovered following initial treatment. Overall, these results suggest the potential future development of these materials as a mucoadhesive drug delivery system for the treatment of diseases of the anterior segment and in particular testing in appropriate animal models of disease.

## Supplemental Material


Supplemental Material - Mucoadhesive micelles for ophthalmic drug delivery
Supplemental Material for Mucoadhesive micelles for ophthalmic drug delivery by Taylor Goostrey, Mitchell Ross, Karim Soliman, Lindsay Sheardown, and Heather Sheardown in Journal of Biomaterials Applications.
