# Supplementary figures and images for: Mucoadhesive micelles for ophthalmic drug delivery
Source: J Biomater Appl. 2025 Oct 7;40(8):972–86. doi: 10.1177/08853282251386004 (PMC12876411; doi:10.1177/08853282251386004)

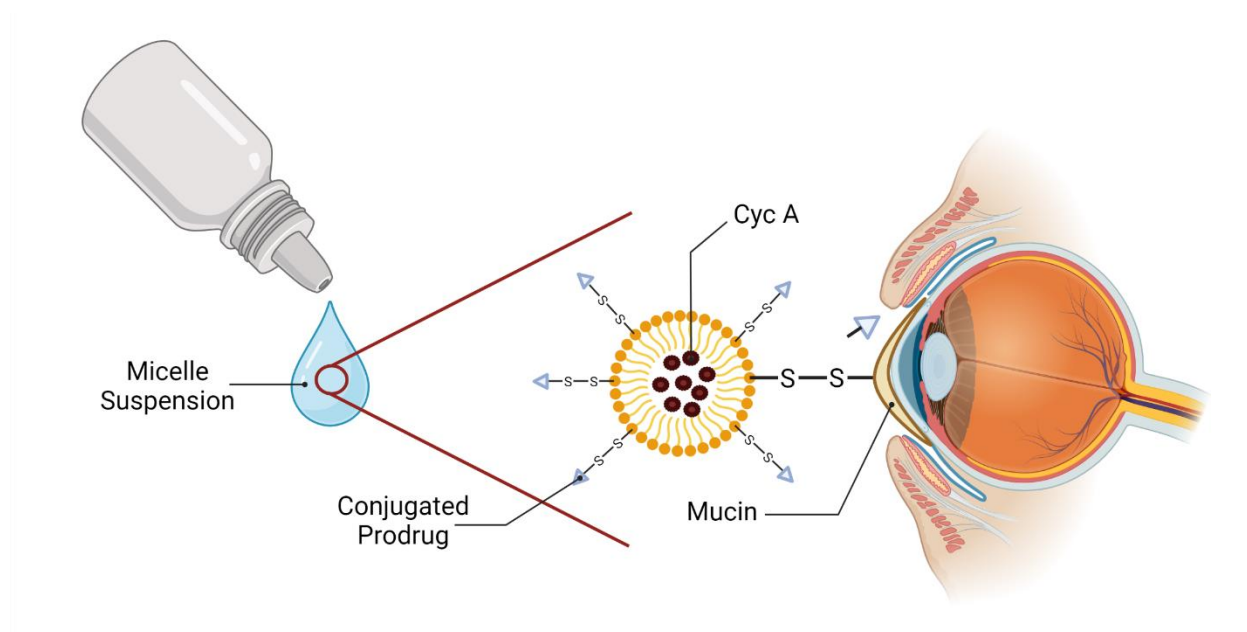

*Graphical Abstract*

Supplement: Supplemental Material - Mucoadhesive micelles for ophthalmic drug delivery [file sj-pdf-1-jba-10.1177_08853282251386004.pdf]
